# Assessment of background dose rate on non-human biota in a Mediterranean terrestrial ecosystem

**DOI:** 10.1007/s11356-024-35292-5

**Published:** 2024-10-26

**Authors:** Javier Guillén, Almudena Real, Alejandro Salas, Danyl Pérez, Juan Gabriel Muñoz-Muñoz, Alicia Escribano, Agustina Sterling

**Affiliations:** 1 LARUEX, Facultad de Veterinaria, Universidad de Extremadura, Avda. Universidad, s/n, 10003 Cáceres, España; 2 CIEMAT, Radiation Protection of the Public and the Environment, CIEMAT, Avda. Complutense, 40, 28040 Madrid, Spain; 3 CSN, Nuclear Safety Council, c/ Pedro Justo Delgado Dellmans, 11, 28040 Madrid, Spain

**Keywords:** RAP, Dose rate, ERICA Tool, Non-human biota, Mediterranean

## Abstract

The assessment of radiological impact to the environment is usually carried out by the dose rate estimation to hypothetical entities named Reference Animals and Plants (RAPs). There are many codes to carry out this assessment, which requires the definition of a scenario and using site-specific transfer parameters when possible. Transfer parameters present a geographical bias, as they are mostly derived from temperate and arctic climate datasets, but there is a scarcity of data for Mediterranean climates. In this study, a terrestrial Mediterranean scenario was defined using the distribution of activity concentrations of anthropogenic (^90^Sr, ^137^Cs) and naturally occurring radionuclides (^40^ K, ^210^Pb, ^210^Po, ^226,228^Ra, ^235,238^U, ^232^Th) in Cáceres province (Spain). Site-specific transfer factors, CR_wo-media_, defined as the ratio between the concentration in the whole organism and the medium (soil in this case) were considered. Dose rate assessments for terrestrial RAPs were carried out using Tier 3 in ERICA Tool, ranging 0.23–3.73 µGy/h which is below the screening level of 10 µGy/h. Therefore, no harmful effects are expected to occur. Internal dose rate predominates over external one because the main contributors are naturally occurring radionuclides (in most cases ^40^ K, ^226,228^Ra, ^210^Pb, ^210^Po), which are mostly α-emitting radionuclides. These results can be used for the evaluation of other radiological and nuclear installations in Mediterranean climates, as they set the background dose rate.

**Supplementary Information:**

The online version contains supplementary material available at 10.1007/s11356-024-35292-5.

## Introduction

Radiological protection of the environment evolved from an anthropogenic perspective to the recommendation to be demonstrated *per se* (ICRP 2007). In order to do so, hypothetical entities named Reference Animals and Plants (RAPs) were defined considering a similar methodology as that used in human radiological protection with the reference person (ICRP 2008). These RAPs are defined at the family level, with given life span and anatomic and physiological characteristics, for different ecosystems (terrestrial, freshwater, and marine). There are many numerical codes available to assess the radiological impact on non-human biota via the estimation of the total dose rate for the different organisms in a given ecosystem or scenario: ERICA Tool (Brown et al. 2008, 2016); RESRAD-BIOTA (USDoE 2002) and R&D128/SP1a (Copplestone et al. 2001, 2003), ECOMOD (Sazykina 2000), EDEN 2 (Beaugelin-Seiller et al. 2006), K-Biota (Keum et al. 2011), LIETDOS-BIO (Nedveckaite et al. 2007), PC-CREAM 08 (Anderson et al. 2022), D-DAT (Beaugelin-Seiller et al. 2006), CROMERICA (Mora et al. 2015), NRCDose (USNRC 2020). The estimated dose rates are compared to Derived Consideration Reference Levels (DCRLs), which can be considered dose rate bands within which there is likely to be some chance of deleterious effects being observed in the organisms (ICRP 2008). Thus, reference/screening levels below which no harmful effect is expected to occur were established. The benchmark dose rates were considered 40 μGy/h for terrestrial animals, birds, amphibians, and reptiles, and 400 μGy/h for other aquatic organisms, which are considered to be protective of populations of non-human biota (IAEA 1992; UNSCEAR 1996; US DoE, 2002). Additionally, ERICA Tool has also a screening level of 10 μGy/h (Brown et al. 2008, 2016).

As there are many codes to assess the dose rate for non-human biota, there have been extensive intercomparisons of various codes conducted under the auspices of the IAEA (Vives i Batlle et al. 2007; Beresford et al. 2008a, b; Beresford et al. 2010; Vives i Batlle et al. 2011). Comparing the results provided by two of the most used (ERICA Tool and RESRAD-BIOTA), it may be concluded that, although the numerical results were not the same, they were of similar order of magnitude and reached the same conclusions regarding whether or not there were adverse effects and which radionuclides were the main contributors to the dose rate (Andersson et al. 2017; Cujić and Dragović 2018; Sotiropoulou and Florou 2020). However, it must be taken into account that ERICA has a bigger database of radionuclides and reference organisms than RESRAD-BIOTA (Brown et al. 2008, 2016; USDoE 2002).

The importance of using site-specific transfer parameters was also pointed out in another intercomparison (Wood et al. 2009). These transfer parameters are usually considered concentration ratios (CR_wo-media_) between the element/activity concentration in the whole organism (wo) and the media. There are international compilations for CR values in terrestrial, freshwater, and marine ecosystems (Copplestone et al. 2013; IAEA 2014). The CR values are reported on an element basis, without isotope differences. Although some differences between CR values obtained from radioisotope and stable element data were reported, with mean values for ^137^Cs:stable Cs and ^90^Sr:stable Sr ratios reported as 1.54 and 35, respectively, for grass (Guillén et al. 2022), CR values for stable elements can be used for estimation purposes if no further data are available. However, there are many radionuclide-organism-ecosystem combinations for which no data is available, so some extrapolation approaches were considered within the ERICA Tool (Brown et al. 2013; Beresford et al. 2016). A geographical bias was also noticed, as most of the reported data come predominantly from North Europe, Japan, North America, and Australasia, and mainly in temperate and arctic ecosystems (Howard et al. 2013).

Occasionally, the dose assessment to other organisms not included in ICRP’s RAPs list may be useful, e.g., an endangered species. Thus, other types of animals and plants can be also introduced in the codes (ERICA Tool and RESRAD-BIOTA), defining their dimensions (usually an ellipsoid), and occupancy factors, which is defined as the fraction of the time that an organism spends at a specified position in its habitat. In this way, dose rates to fungi in Spanish ecosystems (Guillén et al. 2017), ants in Swedish forest ecosystems (Rosén et al. 2018), sheep in Greece (Sotiropoulou et al. 2017), marine and terrestrial biota in Antarctic (Szufa et al. 2020), bear in Croatia and Poland (Skoko et al. 2023), wild boars in Germany (Stager et al. 2023), and numerous organisms (hare, fox, very small birds) in several scenarios in Belgium (Vives i Batlle et al. 2016; Sweeck et al. 2024) have been estimated. In these cases, CR values may be extrapolated from other organisms already reported in the bibliography (Brown et al. 2013; Beresford et al. 2016) or were obtained experimentally and are therefore, site-specific (Vives i Batlle et al. 2016; Guillén et al. 2017; Rosén et al. 2018). Another option is the use of archive samples (Szufa et al. 2020; Skoko et al. 2023). Determination of whole organism activity concentration may pose a problem, since these archive samples consist mainly of bones or part of the organisms, not whole-body samples. However, they can be estimated using reported whole-body tissue ratios (Yankovich et al. 2010; IAEA 2014). Then, CR values can be also estimated using radionuclide concentrations in soil from aerial deposition data in case of any radioactive fallout.

The main goal of the study is the estimation of the total, external and internal dose rate for terrestrial RAPs in a Mediterranean ecosystem, to be used as background dose rate for further radiological environmental assessment for non-human biota affected by radiological or nuclear installations or other scenarios involving NORM (Naturally Occurring Radioactive Material) materials in Spain. In this study, a scenario in a Mediterranean climate was defined using as input data the distribution of naturally occurring and anthropogenic radionuclides in soil for the Cáceres province (Extremadura, Spain) (Baeza et al. 1993, 1994) obtained in previous studies, and the CR_wo-media_ values obtained for ICRP RAPs in a Mediterranean ecosystem in the same province (Guillén et al. 2018). Thus, the incremental value in the RAP’s dose rates for those scenarios, e.g., impact of a NPP (nuclear power plant), can be estimated just by subtracting the background value.

## Material and methods

### Definition of a Mediterranean climate scenario

In order to define a Mediterranean climate scenario, the Extremadura region was selected as it was shown previously (Guillén et al. 2018) that it complies with the “Csa” climate in the Köppen classification (Kottek et al. 2006, AEMET 2018), with an annual average temperature of 16 °C and hot summers. Soil activity concentrations used as input data for the dose rate assessment are given in Table 1, corresponding to previous studies about the distribution of anthropogenic and naturally occurring radionuclides in Cáceres province (Baeza et al. 1993, 1994), part of Extremadura and with an extension of 19,868 km^2^ (2^nd^ largest in Spain). The use of mean values and distribution functions from these data takes into account the possible spatial heterogeny of the deposited radionuclides. A layer of soil of 0–10 cm was considered following international recommendations (IUR 1992). As the main source of anthropogenic radionuclides in the considered ecosystem was global fallout, only ^137^Cs and ^90^Sr were considered; the activity concentration of ^239+240^Pu and ^241^Am were much lower in this ecosystem (Baeza et al. 2006; Guillén et al. 2015). Assuming soil bulk density to be 1.6 g/cm^3^ (Rubio-Delgado et al. 2017), and decay correcting these values to the present date (2024), the mean value and range of the activity concentration of ^137^Cs and ^90^Sr in soils (0–10 cm) in Extremadura were derived (see Table 1). Due to the low level of the activity concentration for ^137^Cs, no additional corrections considering exponential distribution with depth were undertaken. Thus, this assumption can be considered as conservative. ^137^Cs was originally determined by γ-spectrometry, and ^90^Sr by gas-flow proportional counter after radiochemical separation (Baeza et al. 1993).

**Table 1:** Mean value and range of the activity concentration used as input data in the dose rate assessment in the considered Mediterranean scenario. ^238^U and ^210^Pb were derived from ^235^U and ^226^Ra data, respectively (see “Definition of a Mediterranean climate scenario”)

Radionuclide	Number	Activity concentration (Bq/kg d.m.)	Distribution	Reference
^137^Cs	199	1.39 (0.10–14.5)	Log-normal	Baeza et al. (1993
^90^Sr	31	0.63 (0.07–2.25)	Log-normal	
^226^Ra	263	38.3 (13–165)	Log-normal	Baeza et al. (1994
^235^U	263	3.1 (0.7–10.4)	Log-normal	
^238^U	–-	68 (15.6–231.1)	Log-normal	
^232^Th	263	41 (7–204)	Log-normal	
^40^ K	263	653 (48–1586)	Normal	
^210^Pb	–-	54 (28.6–181)	Log-normal	Barrera et al. (2008

Naturally occurring radionuclides (^226^Ra, ^235^U, ^232^Th, and ^40^ K) depth distributions in soil are usually homogeneous in vertical soil profiles, and it was considered as such in this scenario. These radionuclides were originally determined by γ-spectrometry after waiting for secular equilibrium of ^226^Ra and its daughters to be reached (Baeza et al. 1994). Quality control of these measures was made through satisfactory participation in national and international proficiency tests and the use of reference materials, such as Soil 6 from IAEA. The atmospheric contribution of ^210^Pb deposition in soil was estimated to be about 1000 Bq/m^2^ (dry component) and 3 Bq/m^2^ per each annual rainfall mm (wet component) (Barrera et al. 2008). Considering a mean value of 500 mm for the Cáceres province (AEMET 2018), the unsupported ^210^Pb can be estimated in 2500 Bq/m^2^, which using the same soil bulk density and considerations as for ^137^Cs and ^90^Sr can be estimated to be 15.6 Bq/kg in the 0–10 cm soil layer. The total concentration of ^210^Pb in soil was the sum of the unsupported contribution and that in equilibrium with ^226^Ra (supported). In this scenario, the concentration of ^238^U was derived from that of ^235^U using the ^235^U/^238^U ratio for natural uranium, which is the case of this region; and ^238^U and ^234^U were assumed to be in secular equilibrium. ^232^Th and ^228^Ra, and ^210^Pb and ^210^Po were also considered to be in secular equilibrium in this scenario. The contribution of radon and thoron was not considered in this study because of the lack of data on soil concentrations of these radionuclides. Beresford et al. (2012) analyzed their contribution to different burrowing animals, as usual concentration in soil pore is in the range of kBq/m^3^, providing mean internal dose rates in the range of 1 to a few tens of µGy/h. Their contribution to other RAPs on soil or above soil may be considered negligible due to the dilution effect of open air on radon and thoron concentration, usually in the range of 1–10 Bq/m^3^.

### Dose rate assessment for non-human biota

The dose rate assessment was carried out with ERICA Tool 2.0 (Brown et al. 2008, 2016) using the probabilistic functionality of Tier 3 with 10,000 simulations for each RAP considered. Weighted dose rates were estimated using the default radiation weighting factors from the ERICA Tool of 10 for α, 3 for low energy β, and 1 for other β and γ emissions. Default occupancy factors were considered.

Transfer factors (CR_wo-media_) are defined as the ratio between the equilibrium activity concentration of a radionuclide in an organism and the corresponding medium (ICRP 2009) (Eq. 1). In the existing models and data compilations applied in environmental impact assessments, CR_wo-media_ values are presented by element assuming the same value for all isotopes (of that element) including stable isotopes (Eq. 2) (Beresford et al. 2008a, b; Copplestone et al. 2013; IAEA 2014):

1 $${CR}_{wo-media}=\frac{\mathrm{Activity}\;\mathrm{concentration}\;\mathrm{radionuclide}\;X\;in\;whole\;body\;RAP\;\left(Bq/kg\;FM\right)}{Activity\;concentration\;radionuclide\;X\;in\;soil\;\left(Bq/kg\;DM\right)}$$

2 $${CR}_{wo-media}=\frac{Concentration\;element\;X\;in\;whole\;body\;RAP\;\left(mg/kg\;FM\right)}{Concentration\;element\;X\;in\;soil\;\left(mg/kg\;DM\right)}$$

Table 2 shows the mean value and range of CR_wo-media_ values used in the ERICA Tool assessments. Data were mainly from (Guillén et al. 2018) corresponding to site specific of the Mediterranean climate, but if no data were available, values reported in IAEA TRS479 (IAEA 2014) and ERICA Tool 2.0 database were used. The values CR_wo-media_ for Ra for the Mediterranean climate were considered as those reported for Ba (Guillén et al. 2018). The rationale behind this consideration is based on the fact that both elements are alkaline-earth, and therefore with similar chemical behavior. In fact, ^133^Ba is used as an internal spike for the determination of ^226^Ra in radiochemical separations (Baeza et al. 1998). This approach must be checked when considering scenarios with NORM residues, as the total dose rate originated from ^226^Ra might be underestimated.

**Table 2:** Transfer parameters (CR_wo-media_) for the different RAPs used in the Mediterranean climate scenario

Element	Wild grass	Earthworm	Bee	Frog	Duck	Rat	Deer	Pine tree
Cs	0.088(0.004–0.41)	0.0033(0.0023–0.0050)	0.028(0.024–0.0031)	0.021(0.0063–0.040)	0.54**	0.044(0.0057–0.12)	0.0063(0.0023–0.011)	0.0018(0.0005–0.0031)
K	1.73(0.13–7.16)	0.19(0.14–0.23)	2.55(2.43–2.7)	0.46(0.44–0.48)	0.22	0.72(0.43–0.93)	0.53(0.49–0.56)	0.22(0.014–0.027)
Pb	0.13(0.0058–0.63)	0.025(0.011–0.053)	0.018(0.0094–0.029)	0.0049(0.0020–0.011)	0.046**	0.0018(0.00086–0.0043)	0.012(0.0032–0.021)	0.0021(0.0015–0.0029)
Po	0.31(0.017–1.90)*	0.10*	0.076**	0.087**	0.088**	0.087**	0.086(0.00024–1.10)*	0.038(0.013–0.055)*
Ra (Ba)	0.35(0.019–0.80)	0.0030(0.0019–0.0037)	0.083(0.072–0.090)	0.36(0.029–0.041)	0.20**	0.051(0.0082–0.22)	0.19(0.14–0.21)	0.0011(0.0008–0.0014)
Sr	1.18(0.084–3.7)	0.071(0.050–0.11)	0.27(0.24–0.28)	0.52(0.47–0.56)	0.97**	0.53(0.14–1.86)	0.92(0.62–1.09)	0.016(0.011–0.024)
Th	0.24(0.00022–2.7)*	0.011**	0.0029**	0.00057*	0.00057**	0.0011**	0.00014(0.000013–0.00064)*	0.0011(0.00001–0.0031)*
U	0.031(0.018–0.041)	0.014(0.0063–0.024)	0.011(0.0006–0.0018)	0.00095(0.00041–0.0019)	0.00045**	0.00018(0.00010–0.00027)	0.00020(0.000086–0.00032)	0.0068(0.000014–0.032)*

CR_wo-media_ values for Ra were considered equal to those reported for Ba (see “Dose rate assessment for non-human biota”). It should be noted that ERICA Tool 2.0 does not provide CR values for RAPs explicitly, but for more generic organism groupings: (amphibian = frog, annelid = earthworm, bird = duck, flying insects = bee, grass and herbs = grass, mammal large = deer, mammal small burrowing = rat, tree = pine tree)

^*^Data from IAEA TRS479 (IAEA 2014)

^**^Data from ERICA Tool database (Brown et al. 2016), otherwise data from (Guillén et al. 2018)

## Results

Table 1 shows the mean value and range of the activity concentration for ^137^Cs, ^90^Sr, ^226^Ra, ^235^U, ^238^U, ^232^Th, ^40^ K, and ^210^Pb in the layer of soil (0–10 cm) in the province of Cáceres (Spain), classified as Mediterranean climate in a previous study (Guillén et al. 2018). The activity concentration of ^137^Cs and ^90^Sr can be considered low, because the global fallout was the main source of anthropogenic radionuclides in this region, reflecting the fact that the influence of Chernobyl or Palomares accidents was very limited (De Cort et al. 1998; Sancho and García-Tenorio 2019). The distribution was considered log-normal for all radionuclides, except for ^40^ K, which was demonstrated normal (Baeza et al. 1994). Table 2 shows the mean value and range of CR_wo-media_ for Cs, K, Pb, Po, Ra, Sr, Th, and U used in the assessment. Whenever possible, site-specific values were considered (Guillén et al. 2018), except for RAP duck because data for only one individual were reported in a previous study (Guillén et al. 2018), and CR values were taken from the literature (IAEA 2014; Brown et al. 2016).

The results of the assessment for the different RAPs considered (wild grass, earthworm, bee, frog, duck, rat, deer, and pine tree) in the Mediterranean climate scenario are presented in Table 3 as mean values and range (5^th^–95^th^ percentile) for external, internal, and total dose rates. It also gives the total dose rate estimation using specific CR values for the Mediterranean ecosystem (see Table 2), and default CR values in ERICA Tool 2.0 (see Table S1 in Supplementary Material). It should be noted that there are no default CR values for ^40^ K. Therefore, total dose rates using default CR are usually lower than those from specific CRs. Regarding earthworm and frog total dose rates from specific CR are lower than those from default CRs, because the specific CR for Pb and Ra (earthworm), and Pb (frog) are 1–2 orders of magnitude lower than default ones.

**Table 3:** Mean value and range (5^th^–95^th^ percentile) of external, internal and total dose rates, expressed in µGy/h, for the different terrestrial RAPs in the considered scenario in Mediterranean climate conditions, using specific CR values from Table 2, and default CR values in ERICA Tool 2.0, without considering the contribution of ^40^ K (no default data)

RAP	Dose rate (µGy/h)			
	Specific CR			Default CR
	External	Internal	Total	Total
Wild grass	0.041 (0.037–0.045)	2.68 (1.95–3.69)	2.72 (1.96–3.73)	2.25 (2.04–2.53)
Earthworm	0.11 (0.10–0.12)	0.70 (0.47–1.02)	0.81 (0.57–1.14)	2.06 (1.10–3.68)
Bee	0.034 (0.033–0.039)	0.90 (0.76–1.09)	0.94 (0.79–1.13)	0.53 (0.47–0.62)
Frog	0.11 (0.10–0.12)	0.49 (0.30–0.88)	0.56 (0.41–1.00)	1.54 (0.36–4.28)
Duck	0.041 (0.037–0.045)	0.35 (0.29–0.42)	0.39 (0.33–0.47)	0.33 (0.30–0.37)
Rat	0.11 (0.096–0.12)	0.90 (0.74–1.09)	1.00 (0.83–1.20)	0.82 (0.74–0.91)
Deer	0.029 (0.026–0.031)	0.89 (0.75–1.05)	0.91 (0.78–1.08)	0.78 (0.71–0.87)
Pine tree	0.034 (0.031–0.037)	0.27 (0.20–0.35)	0.30 (0.23–0.39)	0.28 (0.24–0.33)

Total dose rates for all RAPs are below the ERICA screening level of 10 µGy/h, and the benchmark defined by IAEA of 40 μGy/h (IAEA 1992). Similar results of the total dose rates were reported for deer and grass in Greece (Sotiropoulou et al. 2016), which can also be considered Mediterranean climate, were also in the range of 0.073–0.76 and 0.018–0.47 µGy/h, respectively, and below those screening levels. In other studies, designed to establish a baseline or background dose rate in different climates (Manigandan and Shekar 2018; Petrović et al. 2018; Babić et al. 2020), total dose rates were also below screening levels. Table 4 shows the total dose rate reported in several studies worldwide for different scenarios. Therefore, in a Croatian NORM disposal site (coal ash and slag), an increase in the total dose rate was reported from the background to the disposal areas, although in both areas it was below 10 µGy/h (Skoko et al. 2019). Karimullina et al. (2018) also reported an increase of the total dose rate from ^90^Sr, ^137^Cs, ^239.240^Pu to grass in the East-Ural Radioactive Trace (EURT) from the background to the impact area. In the case of planned exposure scenarios, the total dose rates estimate to non-human biota usually consider the release of anthropogenic and/or naturally occurring radionuclides prone to be released, resulting in dose rates below screening levels in the scenarios considered in Table 4 (Vives i Batlle et al. 2016; Monged et al. 2020).

**Table 4:** Total dose rate, expressed in µGy/h, reported in the literature for terrestrial RAPs

Country	Scenario	Radionuclides considered	RAPs	Total dose rate (µGy/h)	Reference
Antartic	Existing exposure	^90^Sr, ^137^Cs, ^238,239.240^Pu, ^241^Am	Grass	0.007	Szufa et al. (2020)
Belgium	Existing exposure (contaminated catchment), conservative assessment	Anthropogenic and naturally occurring	Frog*	55.7	Sweeck et al. (2024)
			Earthworm*	61.8	
			Duck*	10.8	
			Bee*	15.3	
			Grass*	48.6	
			Deer*	25.2	
			Rat*	26.4	
			Pine tree*	57.4	
	Planned exposure (low level radioactive waste disposal site, wetlands)	Anthropogenic and naturally occurring	Frog*	0.020	Vives i Batlle et al. (2016)
			Earthworm*	0.038	
			Duck*	0.00017	
			Bee*	0.037	
			Grass*	0.00045	
			Deer*	0.00025	
			Rat*	0.00041	
			Pine tree*	0.00017	
Croatia	Existing exposure	^238^U, ^226^Ra, ^210^Pb, ^232^Th, ^40^ K, ^134^Cs, ^137^Cs	Frog*	1.2	Babić et al. (2020)
			Earthworm*	1.2	
			Duck*	0.7	
			Bee*	1.1	
			Grass*	6.6	
			Deer*	1.2	
			Rat*	1.2	
			Pine tree*	0.6	
	NORM Disposal site	^235,238^U, ^226^Ra, ^210^Pb, ^232^Th	Pine Tree*	0.5–1.19	Getaldić et al. (2023)
	NORM Disposal site	^226^Ra, ^232^Th, ^137^Cs	Frog*	0.41	Petrović et al. (2018)
			Earthworm*	0.41	
			Duck*	0.32	
			Bee*	0.35	
			Grass*	1.53	
			Deer *	0.54	
			Rat *	0.57	
			Pine tree*	0.12	
	NORM Disposal site	^235,238^U, ^226^Ra, ^210^Pb, ^232^Th, ^40^ K	Grass (background area)	1.3	Skoko et al. (2019)
			Grass (disposal area)	2.2	
			Tree (background area)	0.5	
			Tree (disposal area)	0.8	
Egypt	Planned exposure	Anthropogenic expected to be emitted in routine operation of a NPP	Frog*	0.0036	Monged et al. (2020)
			Earthworm*	0.0027	
			Duck*	0.0036	
			Bee*	0.0028	
			Grass*	0.0034	
			Deer*	0.0036	
			Rat*	0.0036	
			Pine tree*	0.0036	
Finland	Existing exposure	^134,137^Cs	Deer	0.39–1.79	Vetikko and Kostiainen (2013)
Greece	Existing exposure	^226,228^Ra, ^228^Th, ^134,137^Cs, ^131^I	Grass	0.018–0.057	Sotiropoulou et al. (2016)
			Deer*	0.073–0.76	
	Existing exposure	^226,228^Ra, ^228^Th, ^137^Cs	Grass	0.47	Sotiropoulou and Florou (2020)
			Deer*	0.52	
India	Existing exposure	^238^U^a^, ^210^Po, ^232^Th	Grass	0.35–1.22^a^	Manigandan and Shekar (2018)
Korea	NORM waste stockyard	^238^U, ^226^Ra, ^210^Pb, ^210^Po, ^232^Th, ^40^ K	Frog*	~ 1^b^	Lee and Yi. (2023)
			Earthworm *	~ 1^b^	
			Duck*	~ 0.2^b^	
			Bee*	~ 0.2^b^	
			Grass*	~ 1.5^b^	
			Deer*	~ 1^b^	
			Rat*	~ 1^b^	
			Pine tree*	~ 0.1^b^	
Lithuania	Existing exposure	^90^Sr, ^134, 137^Cs, ^54^Mn, ^60^Co, ^238^Pu, ^238^U, ^232^Th,	Frog*	0.063	Konstantinova et al. (2015)
			Earthworm*	1.09	
			Duck*	0.067	
			Bee*	0.0026	
			Grass*	0.51	
			Deer*	1.27	
			Rat*	1.38	
			Pine tree*	0.35	
Nigeria	Gold mining	^234,238^U, ^226^Ra, ^210^Pb, ^210^Po, ^230,232^Th	Frog*	~ 27	Bello et al. (2022)
			Earthworm*	~ 7	
			Duck*	~ 0.5	
			Bee*	~ 25	
			Grass*	~ 5	
			Deer*	~ 0.5	
			Rat*	~ 0.5	
			Pine tree*	~ 0.2	
Russian Federation	EURT	^90^Sr, ^137^Cs, ^239.240^Pu	Grass (background area)	0.0014–0.015	Karimullina et al. (2018)
			Grass (bufer area)	0.097–13.8	
			Grass (impact area)	4.8–158	
Serbia	Coal-fired Power Plant	^234,238^U, ^226^Ra, ^210^Pb, ^210^Po, ^230,234,232^Th, ^137^Cs	Frog*	0.63	Cujić and Dragović (2018)
			Earthworm*	0.52	
			Duck*	0.38	
			Bee*	0.46	
			Grass*	2.93	
			Deer*	0.66	
			Rat*	0.65	
			Pine tree*	0.27	
	Existing exposure	^226^Ra, ^232^Th, ^40^ K	Frog*	0.42	Petrović et al. (2018)
			Earthworm*	0.42	
			Duck*	0.32	
			Bee*	0.35	
			Grass*	0.15	
			Deer*	0.54	
			Rat*	0.57	
			Pine tree*	0.12	

*NORM* naturally occurring radioactive material, *EURT* east-ural radioactive trace, *NPP* nuclear power plant

^*^Reported as organism in ERICA Tool nomenclature (amphibian = frog, annelid = earthworm, bird = duck, flying insects = bee, grass and herbs = grass, mammal large = deer, mammal small burrowing = rat, tree = pine tree)

^a^Total dose rate may be underestimated since ^238^U activity can be assumed to be ^226^Ra taking into consideration the corresponding methodology

^b^Total dose rate not reported, estimated as the visual sum of individual radionuclide contribution

**Fig. 1:**
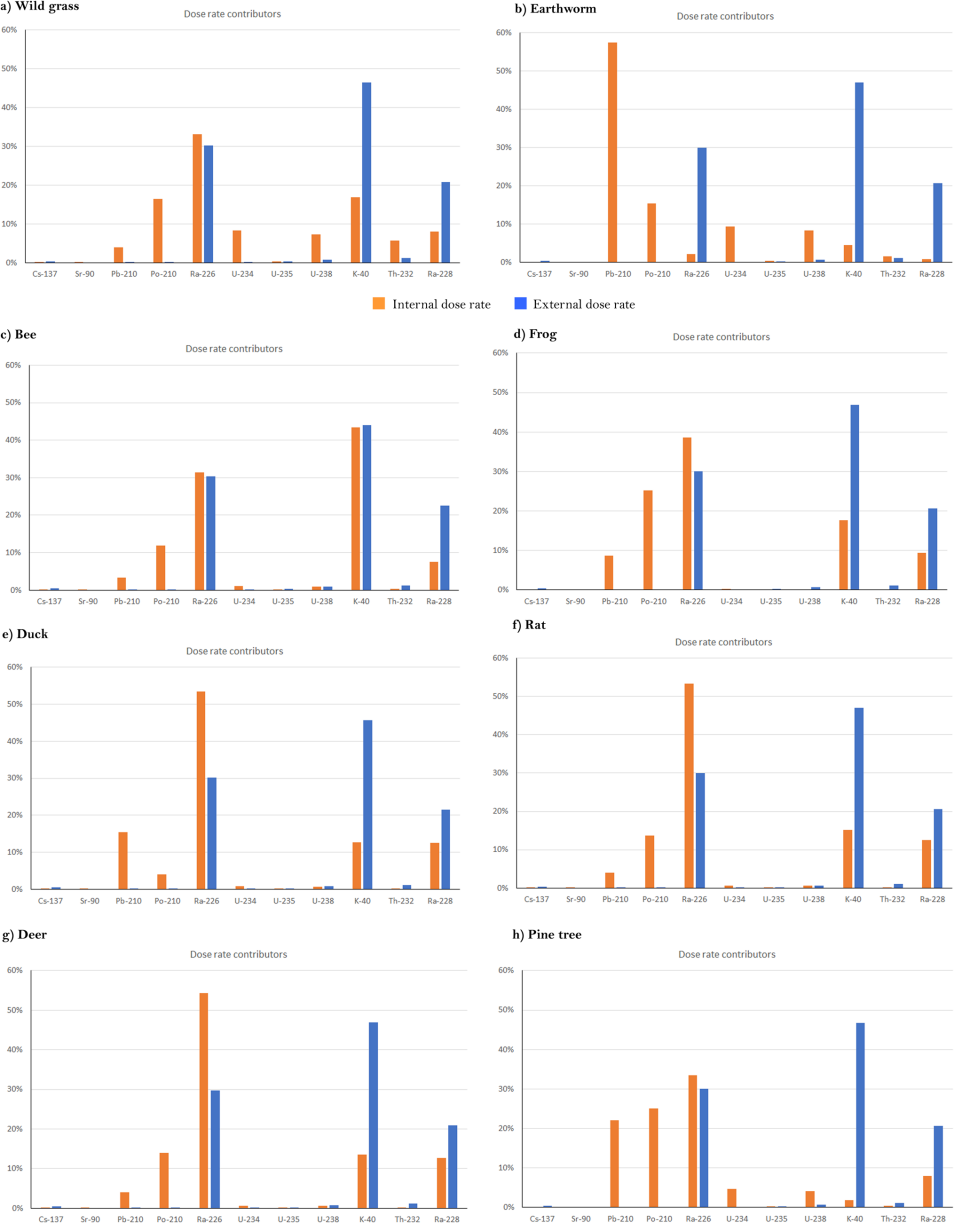
Distribution of the main contributors to the internal and external dose rates, expressed in percentages, for the different terrestrial RAPs in Mediterranean climate conditions: **a** wild grass, **b** earthworm, **c** bee, **d** frog, **e** duck, **f** rat, **g** deer, and **h** pine tree

Regarding the relationship between internal and external dose rates, Table 3 shows that for the Mediterranean climate scenario, the internal dose rate predominates over the external one. This is due to the higher activity concentration of naturally occurring radionuclides, which are mainly α-emitting radionuclides, relative to the low concentration of ^137^Cs and ^90^Sr in soil. Figure 1 shows the contribution of all considered radionuclides to the internal and external dose rates for the terrestrial RAPs. The main contributors to the external dose rate for all RAPs are ^40^K, followed by ^226^Ra and ^228^Ra. Whereas for the internal dose rate, ^226^Ra, ^210^Pb, ^210^Po, ^40^ K, and ^228^Ra are the main contributors, in different percentages depending on the RAP considered. Regarding earthworm, the internal contribution of ^210^Pb was greater than that for ^226^Ra may be due to the fact that ^210^Pb CR value was one order of magnitude higher. The higher contribution of naturally occurring radionuclides to the internal, and therefore to the total dose rate, was also reported by other researchers in different climates (Cujić and Dragović 2018; Petrović et al. 2018; Babić et al. 2020; Sotiropoulou and Florou 2020). The high contribution of naturally occurring radionuclide to the total dose rate can also be inferred from the assessments carried out in NORM disposal or stockyard sites or areas affected by NORM industry activities (coal-fired power plants, gold mining, etc.), as it can be observed in Table 4. Comparing the results obtained in the considered Mediterranean scenario with those reported in Table 4, it can be observed that the background dose rate was higher than those background assessments regarding only anthropogenic radionuclides (not accidental exposure), but similar to those considering naturally occurring radionuclides.

## Conclusions

In this study, a Mediterranean climate scenario was defined in order to assess the background dose rate of terrestrial non-human biota, so that it can be used in further studies to estimate the radiological impact on the environment of nuclear and radiological installations. The scenario was modelled on the basis of the distribution of anthropogenic and naturally occurring radionuclides in the Cáceres province (Spain), using when possible site-specific transfer parameters. The dose rate to terrestrial RAPs (wild grass, earthworm, frog, duck, bee, deer, rat, and pine tree) was in the range 0.23–3.73 µGy/h below screening levels used in ERICA and IAEA, of 10 and 40 µGy/h, respectively. The total dose rate was dominated by the internal one due to the contribution of naturally occurring radionuclides.

## Supplementary Information

Below is the link to the electronic supplementary material.

Supplementary file1 (DOCX 20 KB)

## Data Availability

Radionuclide concentration in Mediterranean soils data used in this study are available at the cited references: Baeza et al. (1993) (10.1007/BF02165071); Baeza et al. (1994) (10.1016/0265-931X(94)90503-7); and Barrera et al. (10.1063/1.2991213). Specific concentration ratios for RAPs in Mediterranean ecosystems data are available at the cited reference: Guillén et al. (2018) (10.1016/j.jenvrad.2017.06.024).
