# Supplementary material for: Assessment of background dose rate on non-human biota in a Mediterranean terrestrial ecosystem
Source: Environ Sci Pollut Res Int. 2024 Oct 26;31(54):62761–71. doi: 10.1007/s11356-024-35292-5 (PMC11599312; doi:10.1007/s11356-024-35292-5)
Supplement: Supplementary file 1 — Supplementary file1 (DOCX 20 KB) [file 11356_2024_35292_MOESM1_ESM.docx]

**Assessment of background dose rate assessment on non-human biota in a Mediterranean terrestrial ecosystem.**

Javier Guillén^a^^[[1]](#footnote-1)^, Almudena Real^b^, Alejandro Salas^a^, Danyl Pérez^b^, Juan Gabriel Muñoz-Muñoz^a^, Alicia Escribano^b^, Agustina Sterling^c^

^a^ LARUEX, Faculty of Veterinary Sciences, University of Extremadura, Avda. Universidad, s/n, 10003, Cáceres, Spain

^b^ CIEMAT, Radiation Protection of the Public and the Environment. CIEMAT. Avda. Complutense, 40, 28040, Madrid, Spain

^c^ CSN, Nuclear Safety Council, c/ Pedro Justo Delgado Dellmans, 11, 28040, Madrid, Spain

Supplementary Material

Table S1

| **Element** | **Wild grass** | **Earthworm** | **Bee** | **Frog** | **Duck** | **Rat** | **Deer** | **Pine tree** |
| --- | --- | --- | --- | --- | --- | --- | --- | --- |
| Cs | 1.13±2.83 | 0.042±0.102 | 0.13±0.47 | 0.45±0.73 | 0.54±1.40 | 2.85±7.82 | 2,85±7,.82 | 0.15±0.31 |
| Pb | 0.11±0.34 | 0.43±1.43 | 0.017±0.033 | 0.046±0.165 | 0.059±0.119 | 0.38±0.136 | 0.038±0.136 | 0.059±0.140 |
| Po | 0.28±0.35 | 0.076±0.021 | 0.076±0.021** | 0.087±0.164** | 0.0997±0.0031* | 0.087±0.164 | 0.087±0.164 | 0.041±0.023 |
| Ra | 0.17±0.37 | 0.20±0.13** | 0.053±0.053 | 0.20±0.23** | 0.035±0.050 | 0.089±0.168 | 0.089±0.168 | 0.017±0.029 |
| Sr | 0.66±1.36 | 0.0614±0.0034 | 0.22±0.98 | 0.97±1.42 | 1.09±2.48 | 1.51±2.67 | 1.51±2.67 | 0.72±1.96 |
| Th | 0.15±0.42 | 0.0112±0.0088 | 0.0030±0.0042* | (5.7±7.1)·10^-4^ | (4.7±9.2)·10^-4^ | 0.0011±0.0043 | 0.0011±0.0043 | 0.0012±0.0015 |
| U | 0.12±0.39 | 0.0341±0.026 | 0.0052±0.0074 | (4.5±5.5)·10^-4^ | 0.0015±0.0018 | 0.0028±0.0056 | 0.0028±0.0056 | 0.0065±0.0014 |

Table S1. Default transfer parameters (CR_wo-media_), mean value ± standard deviation, in ERICA Tool 2.0. It should be noted that ERICA Tool 2.0 does not provide CR values for RAPs explicitly, but for more generic organism groupings: (Amphibian = Frog, Annelid = Earthworm, Bird = Duck, Flying insects = Bee, Grass and herbs = Grass, Mammal large = Deer, Mammal small burrowing = Rat, Tree = Pine tree). * Bayesian approach or methods to derive a missing SD value applied to empirical datasets. ** Similar reference organism

1. Corresponding autor: fguillen@unex.es [↑](#footnote-ref-1)
